# Lung adenocarcinoma concomitant with xeroderma pigmentosum: a case report

**DOI:** 10.1186/s13256-021-02754-0

**Published:** 2021-03-30

**Authors:** Masataka Matsumoto, Kazumi Kaneshiro, Kiyonobu Takatsuki

**Affiliations:** Department of Respiratory Medicine, Kitaharima Medical Center, Ichiba-cho, Ono, Hyogo 675-1392 Japan

**Keywords:** Xeroderma pigmentosum, Lung cancer, Dermatosis, Consanguineous marriage, DNA damage, Ultraviolet rays

## Abstract

**Background:**

Xeroderma pigmentosum is a rare, autosomal-recessive photosensitive dermatosis. Patients with xeroderma pigmentosum have an impaired ability to repair deoxyribonucleic acid damage caused by ultraviolet rays, resulting in skin cancer. Patients with xeroderma pigmentosum are more susceptible to some cancers. We herein report a case of xeroderma pigmentosum accompanied by lung cancer.

**Case presentation:**

The patient was a Japanese woman in her 70s with a family history of consanguineous marriage. Her medical history included squamous cell carcinoma and basal cell carcinoma, in addition to xeroderma pigmentosum. She presented with dry skin with small, pigmented spots, which were particularly focused around the areas exposed to sunlight. Chest computed tomography was conducted to assess for any evidence of metastatic skin carcinoma, and revealed a tumor in the left upper subpleural lobe of the lung. Consequently, she was referred to our department. Finally, we diagnosed lung adenocarcinoma (pT2aN0M1b: stage IVA). She had an epidermal growth factor receptor (EGFR) mutation (p.L858R). Treatment with an epidermal growth factor receptor tyrosine kinase inhibitor (gefitinib) was initiated, and the tumor gradually regressed. No side effects were observed. However, she later died from aspiration pneumonia.

**Conclusions:**

Although xeroderma pigmentosum is rare, a history of consanguineous marriage should be verified. Because of the severe side effects of cisplatin and radiotherapy in xeroderma pigmentosum patients, the risks and benefits of treatment should be considered thoroughly.

## Background

Xeroderma pigmentosum (XP) is an autosomal-recessive, hereditary photosensitive dermatosis. The ability to repair DNA damage caused by ultraviolet (UV) rays is impaired in patients with XP, which frequently results in skin cancer [1]. Steroids maybe useful for many dermatoses, but their utility in the treatment of XP is not established [2, 3].

XP is considered to be rare, with an incidence of 1/22,000 (0.0045%) people in Japan, and 1/1,000,000 (0.0001%) people in Western countries [1]. Although the simultaneous occurrence of XP and lung cancer has been reported previously, lung cancer complications have been reported in only one case to date [4].

Herein, we report a case of lung adenocarcinoma concomitant with XP. The rarity of the case lies in the fact that there was a history of consanguineous marriage (marriage between two individuals that have the same ancestors [5]) and that the patient may not have been treated according to standard guidelines.

## Case presentation

**Fig. 1:**
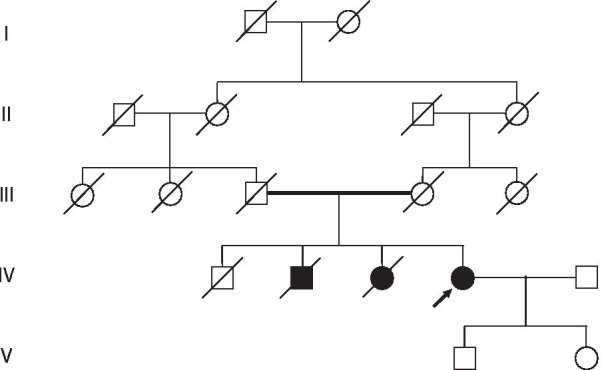
Family tree for this case blotting out xeroderma pigmentosum case. Her paternal and maternal grandmothers were sisters.

**Fig. 2:**
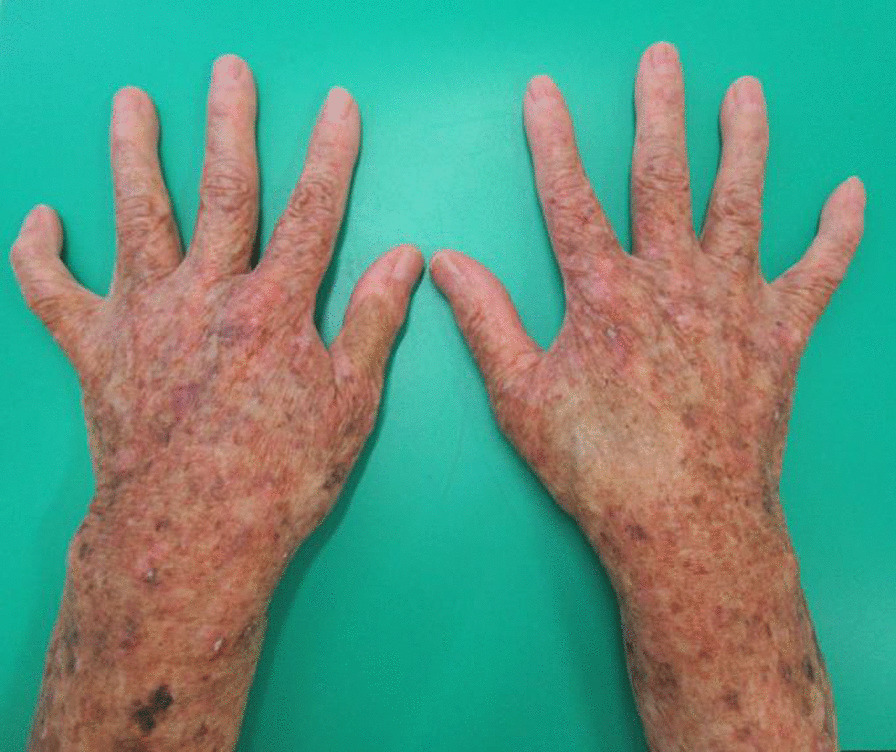
The patient’s hands. Her skin was dry, accompanied by several small, pigmented spots.

The patient was a Japanese woman in her 70s. In 1994, she was diagnosed with an XP variant (XPV) and underwent resection for skin cancer. In the past 10 years, she underwent resection surgeries for the following conditions: squamous cell carcinoma (SCC) of the lower lip (2009); SCC of the upper lip (2011); recurrent SCC of the lower lip (2014); and basal cell carcinoma (BCC) of the lower right eyelid, right earlobe, right forearm, and neck (2018) at the Department of Plastic and Reconstructive Surgery at our hospital. In addition to skin cancer, she had diabetes mellitus (DM) and hypertension. She also underwent the following procedures: an artificial joint replacement surgery for left knee osteoarthritis (2014), endoscopic sphincterotomy for removal of common bile duct stones (2015), laparoscopic cholecystectomy for gallbladder stones (2016), bilateral intraocular lens insertion for cataracts in both eyes, and left external beak incision enforcement for glaucoma due to left exfoliation syndrome (2017). She had two children; the daughter was delivered vaginally, and the son was delivered via cesarean section. Her family history included the following: her elder brother had colon cancer; her second brother had XP, DM, and chronic kidney disease; and her elder sister had XP and dementia. The patient and her parents were born in the same agricultural area of Japan. On more detailed interview of the patient and her family, an instance of consanguineous marriage was identified (Fig. 1), that is, her paternal and maternal grandmothers were sisters. Her ancestry did not include any foreigners. She did not smoke or consume alcohol. At presentation, her skin was dry, accompanied by several small, pigmented spots throughout her body. These spots were particularly focused around the areas exposed to sunlight (Fig. 2). Her eyelids and lips were deformed from the previous resections.

Chest computed tomography (CT), which was advised by the Department of Plastic and Reconstructive Surgery at our hospital and performed on 20 April 2018 to assess any evidence of metastatic skin carcinoma, revealed a tumor (26 × 32 mm) with spicula in the left upper subpleural lobe (S1+2) of the lung. Consequently, she was referred to our department on 15 April 2018. Histopathological examination of the biopsied tumor specimens aided a diagnosis of lung adenocarcinoma (clinical stage cT2aN0M0; stage IB). A thoracoscopy with resection of the upper lobe was attempted on 6 July 2018. However, the resection was discontinued on observation of pleural dissemination. Therefore, the staging was revised to pT2aN0M1b (stage IVA lung adenocarcinoma). Genetic analysis of the specimen from pleural dissemination indicated positivity for an epidermal growth factor receptor (EGFR) mutation (p.L858R). Additionally, immunohistochemistry (IHC) revealed a 22C3-PD-L1 tumor proportion score of 5%.

Following the results of various tests, starting 7 August 2018, we prescribed an EGFR tyrosine kinase inhibitor (gefitinib) as a first-line drug according to the Japanese lung cancer guidelines at that time [6], following which, the tumor gradually regressed (11 × 21 mm), as determined by CT. The patient did not experience any side effects. However, she later died from aspiration pneumonia at a rehabilitation hospital for treatment of knee osteoarthritis on 4 October 2020.

## Discussion and conclusion

This is the first reported case of lung adenocarcinoma concomitant with XP in which a history of consanguineous marriage was confirmed.

XP is a rare, autosomal-recessive hereditary photosensitive skin disease that exhibits a high rate of co-occurrence of specific skin conditions and skin cancer. The skin of patients with XP is extremely sensitive to UV radiation because of congenital defects in the deoxyribonucleic acid (DNA) damage repair mechanism. Insufficient shielding and protection from UV radiation can cause severe photosensitivity symptoms, progression of freckle-like pigment abnormalities, and high rates of recurrent skin cancers despite a young age [1].

XP is classified into eight different genetic groups, from A (XPA) to G (XPG) and XPV. XPA through XPG affect the nucleotide excision repair pathway. The causative genes are known for all XPVs. The XPV gene encodes DNA polymerase eta, which catalyzes accurate translesion synthesis, indicating that the XPV gene contributes to tumor suppression in healthy individuals [7]. The clinical features, disease course, and prognosis vary among XPVs. In Japan, XPA is the most prevalent variant, followed by XPV [1, 8]. Several tools are used for the diagnosis of XP, including the photosensitivity test, DNA repair test using cultured fibroblasts, and genetic analysis using peripheral blood- or patient-derived cultured cells [1].

In this case, we believe that the symptoms of XP appeared because of homozygosity consequent to the consanguineous marriage. XP has almost disappeared in Western countries; however, it has a high prevalence in some regions of Japan [5].

In addition to skin cancer, XP causes malignant brain and spinal cord tumors [9–11]; cancers of the lung [4], uterus [12], breast [12], pancreas [13], stomach [14], kidneys [15, 16], and testicles [17]; and leukemia [18]. A previous study identified an association between XP and lung cancer [4]. Binding of benzo(a)pyrene—a carcinogen found in cigarette smoke—to the DNA causes lung cancer in patients with XP due to impaired DNA damage repair pathways [19, 20]. Thus, patients with XP should avoid exposure to tobacco carcinogens. Our patient was a nonsmoker; however, her husband was a current smoker, which may have affected her condition.

The specific treatment for lung cancer with XP is not described in any lung cancer guidelines. Thus, we treated this case as normal lung cancer. This patient had an EGFR mutation and 5% IHC for 22c3-PD-L1. Therefore, we prescribed gefitinib, which was the first-line drug in Japan at that time, according to the Japanese lung cancer guidelines [6]. If the tumor had further increased in size, we planned to test for EGFR T790M using osimertinib [21]. If EGFR T790M had not been detected, any further increase in size may have mandated the prescription of a different anticancer drug.

However, severe side effects due to cisplatin have been reported in cancer patients with XP [22]. Several *in vitro* studies suggest that cisplatin treatment may have enhanced cytotoxic effects on normal cells as well as cancer cells in patients with XP [23–25]. In the treatment of XP patients with lung cancer, if cisplatin is administered, it should be initiated with caution because of its severe side effects. Further, radiotherapy is not recommended for patients with XP-induced BCC because of the possibility of secondary cancers, which would also complicate the treatment of lung cancer with XP [26].

In the present case, we were unable to exclude the possibility that unknown mechanisms, aside from the impairment of DNA repair systems, were present. We emphasize the need for further research to better understand whether XP patients are more susceptible to toxicities from cisplatin and other chemotherapy agents compared with appropriately matched controls without XP.

Therefore, for XP patients with lung cancer, surgery and/or chemotherapy, aside from cisplatin, should be recommended. When multiple cancers are identified in patients with XP, anticancer therapy should be planned cautiously. Additionally, to avoid this condition, consanguinity should be avoided; if not, genetic counseling should be undertaken.

## Data Availability

Not applicable.

## Abbreviations

XP: Xeroderma pigmentosum
EGFR: Epidermal growth factor receptor
UV: Ultraviolet
XPV: Xeroderma pigmentosum variant
SCC: Squamous cell carcinoma
BCC: Basal cell carcinoma
DM: Diabetes mellitus
CT: Computed tomography
IHC: Immunohistochemistry
TKI: Tyrosine kinase inhibitor
